# Supplementary material for: Precise solid-phase synthesis of CoFe@FeOx nanoparticles for efficient polysulfide regulation in lithium/sodium-sulfur batteries
Source: Nat Commun. 2023 Nov 18;14:7487. doi: 10.1038/s41467-023-42941-9 (PMC10657440; doi:10.1038/s41467-023-42941-9)
Supplement: Supplementary file 1 — Supplementary Information [file 41467_2023_42941_MOESM1_ESM.pdf]

Precise solid-phase synthesis of CoFe@FeO_x nanoparticles for efficient polysulfide regulation in lithium/sodium-sulfur batteries

Yanping Chen[1,8], *Yu Yao*[2,8], *Wantong Zhao*[3], *Lifeng Wang*[2], *Haitao Li*[1], *Jiangwei Zhang*[4], *Baojun Wang*[3], *Yi Jia*[5], *Riguang Zhang*[3,*], *Yan Yu*[2,*], *Jian Liu*[1,4,6,7,*].

[1] State Key Laboratory of Catalysis, Dalian Institute of Chemical Physics, Chinese Academy of Sciences, Dalian, Liaoning 116023, China.

[2] Hefei National Research Center for Physical Sciences at the Microscale, Department of Materials Science and Engineering, National Synchrotron Radiation Laboratory, CAS Key Laboratory of Materials for Energy Conversion, University of Science and Technology of China, Hefei, Anhui 230026, China.

[3] State Key Laboratory of Clean and Efficient Coal Utilization, College of Chemical Engineering and Technology, Taiyuan University of Technology, Taiyuan, Shanxi 030024, China.

[4] Science Center of Energy Material and Chemistry, College of Chemistry and Chemical Engineering, Inner Mongolia University, Hohhot 010021, China.

[5] Department of Applied Chemistry and Zhejiang Carbon Neutral Innovation Institute, Zhejiang University of Technology, Hangzhou 310032, China.

[6] DICP-Surrey Joint Centre for Future Materials, Department of Chemical and Process Engineering, and Advanced Technology Institute, University of Surrey, Guildford, Surrey GU2 7XH, UK.

[7] Center of Materials Science and Optoelectronics Engineering, University of Chinese Academy of Sciences Beijing 100049, China.

[8] These authors contributed equally: Yanping Chen, Yu Yao.

*Corresponding authors: zhangriguang@tyut.edu.cn; yanyumse@ustc.edu.cn; jian.liu@surrey.ac.uk

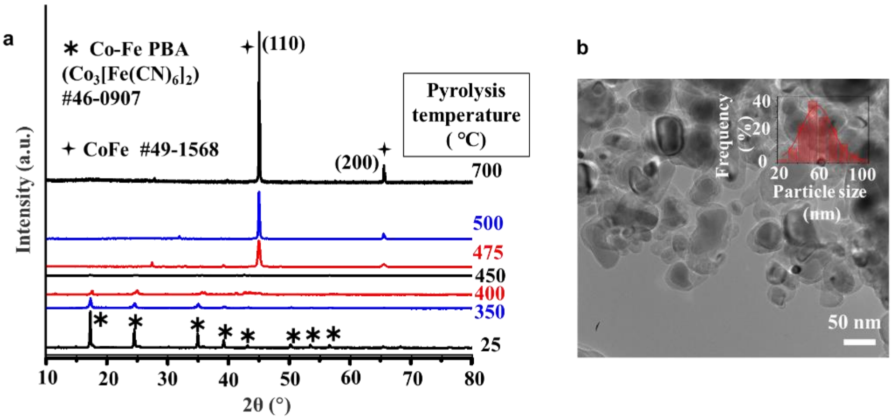

Figure S1 Characterization of Co-Fe PBA and CoFe@C. a XRD patterns of Co-Fe PBA with various pyrolysis temperatures. b The TEM image of the CoFe@C with the size distribution histogram (The iron size distribution is obtained from TEM images analysis by using at least 100 NPs.).

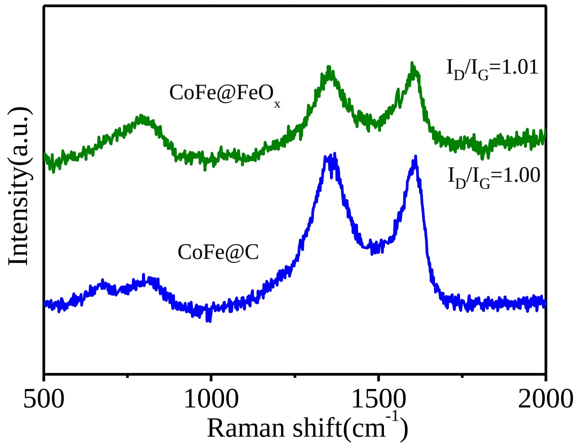

Figure S2 Raman spectra of CoFe@C and CoFe@FeO$_x$.

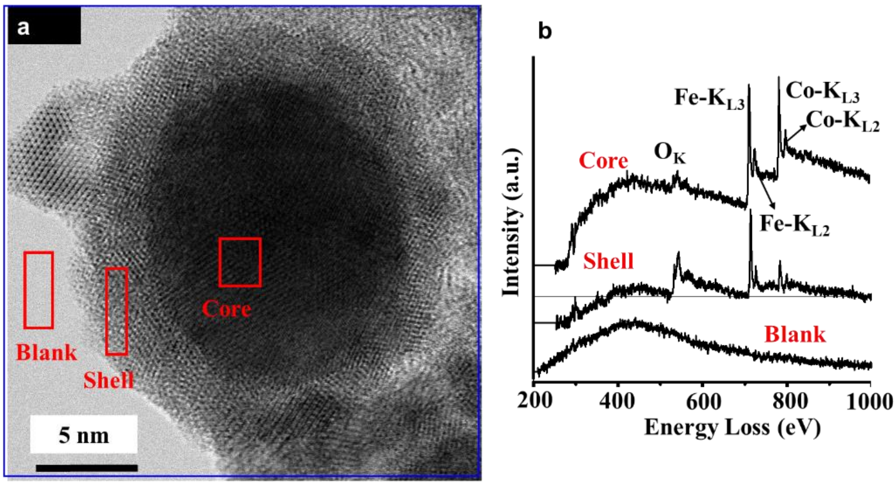

Figure S3 Characterization of CoFe@FeO$_x$. a The STEM image and **b** EELS spectra from locations marked as core, shell and blank of the CoFe@FeO$_x$.

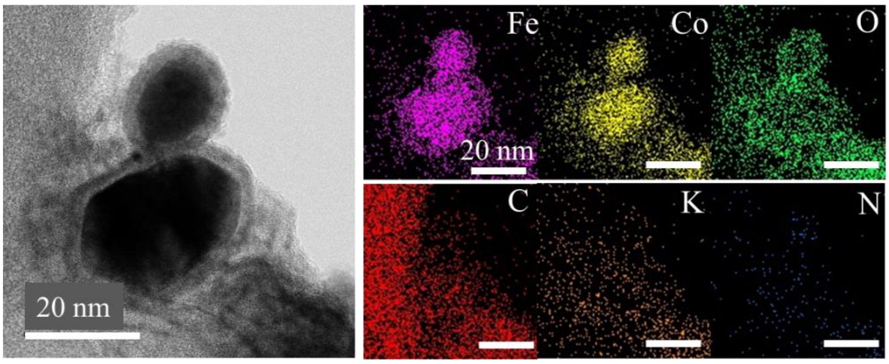

Figure S4 The STEM image and corresponding EDS elemental mapping images of the $CoFe@FeO_x$.

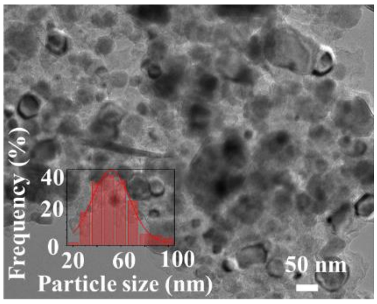

Figure S5 The TEM image of the $CoFe@FeO_x$ with the size distribution histogram (The size distribution is obtained from TEM images analysis by using at least 100 NPs.).

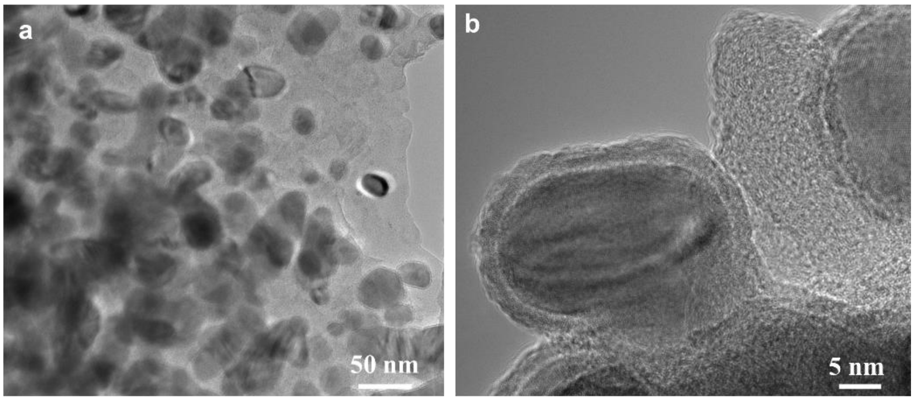

Figure S6 Characterization of $CoFe@FeO_x$. a TEM and **b** HRTEM images of $CoFe@FeO_x$ with syngas treatment temperature at 300 °C.

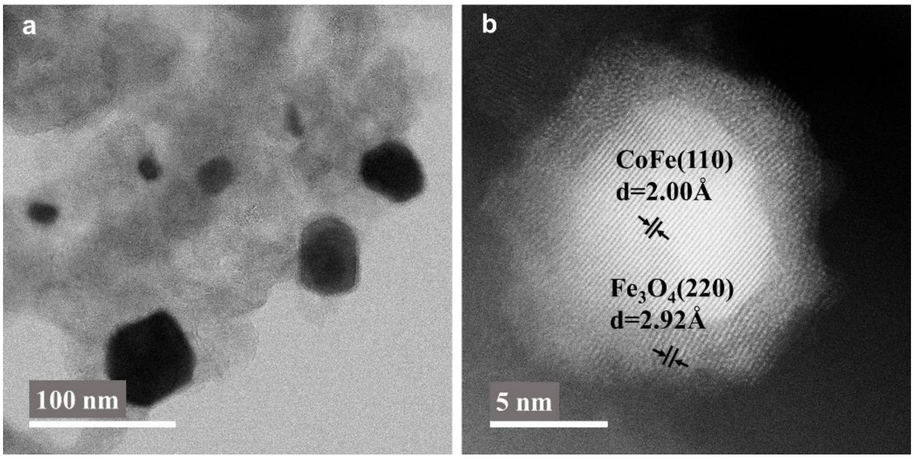

Figure S7 Characterization of CoFe@FeO$_x$. a TEM and **b** HRTEM images of CoFe@FeO$_x$ with syngas treatment temperature at 500 °C.

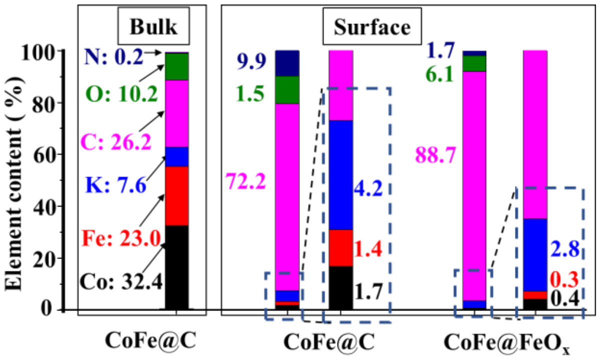

Figure S8 Element content of the bulk (obtained by ICP and element analyzer) and the surface (obtained by XPS) of the CoFe@C and CoFe@FeO$_x$.

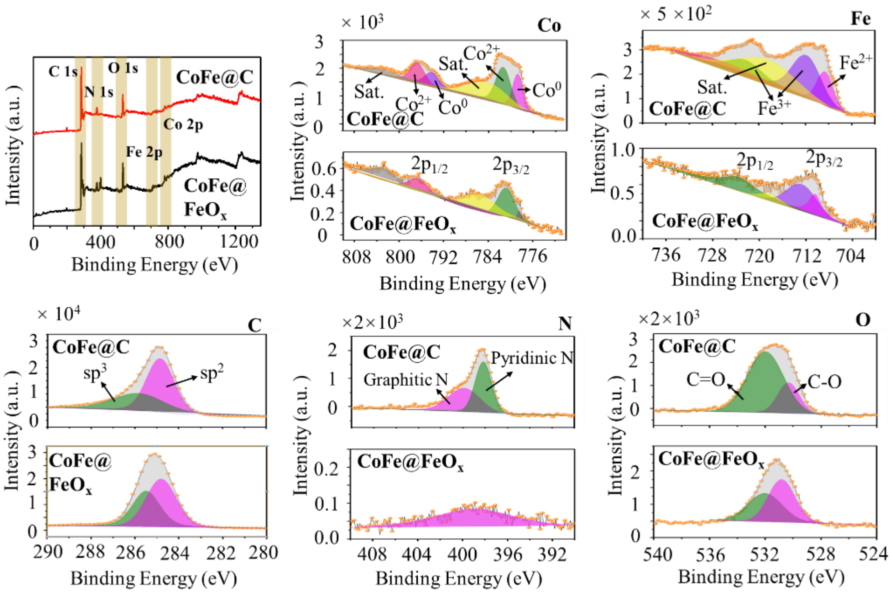

Figure S9 XPS files of the CoFe@C and CoFe@FeO$_x$.

Table S1 Mössbauer parameters of the CoFe@C and CoFe@FeO$_x$

Samples	Assignment	IS (mm s^{-1})	QS (mm s^{-1})	Bhf (T)	Area (%)
CoFe@C	CoFe alloy	0.011	0.00	34.0	96
	FeO$_x$	0.40	0.97	0.0	4
CoFe@FeO$_x$	CoFe alloy	0.012	0	34.19	72
	FeO$_x$	0.26	0.86	0	21
	Fe$_x$C	0.32	0	11.92	7

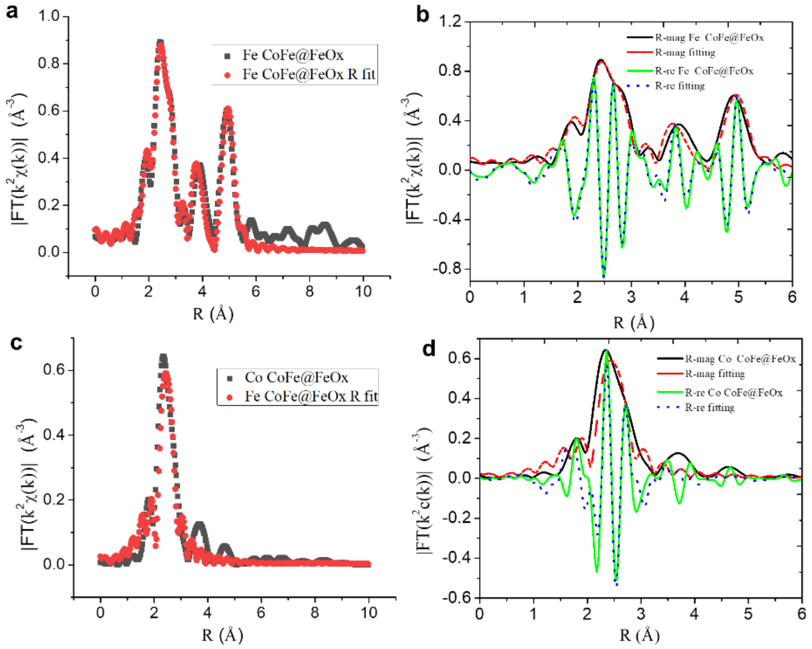

Figure S10 Characterization of CoFe@FeO$_x$. a χ(R) space spectra fitting curve and **b** the inverse FT χ(R) space spectra into χ(q) space spectra (χ(k) space) fitting curve of the CoFe@FeO$_x$ at Fe K edge. **c** χ(R) space spectra fitting curve and **d** the inverse FT χ(R) space spectra into χ(q) space spectra (χ(k) space) fitting curve of the CoFe@FeO$_x$ at Co K edge.

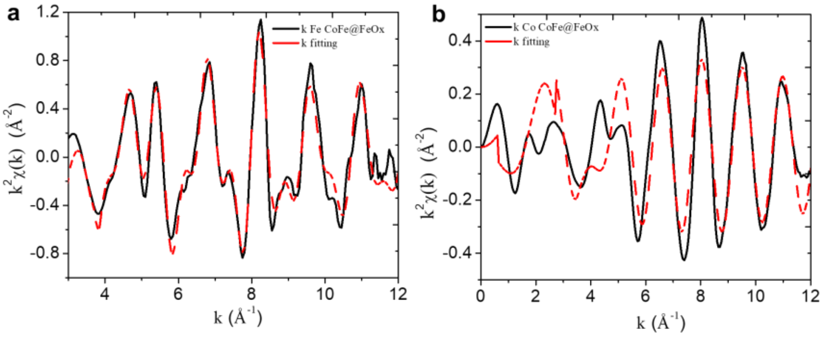

Figure S11 Characterization of CoFe@FeO$_x$. The experimental and fitted data of **a** Fe and **b** Co K-edge EXAFS of the CoFe@FeO$_x$.

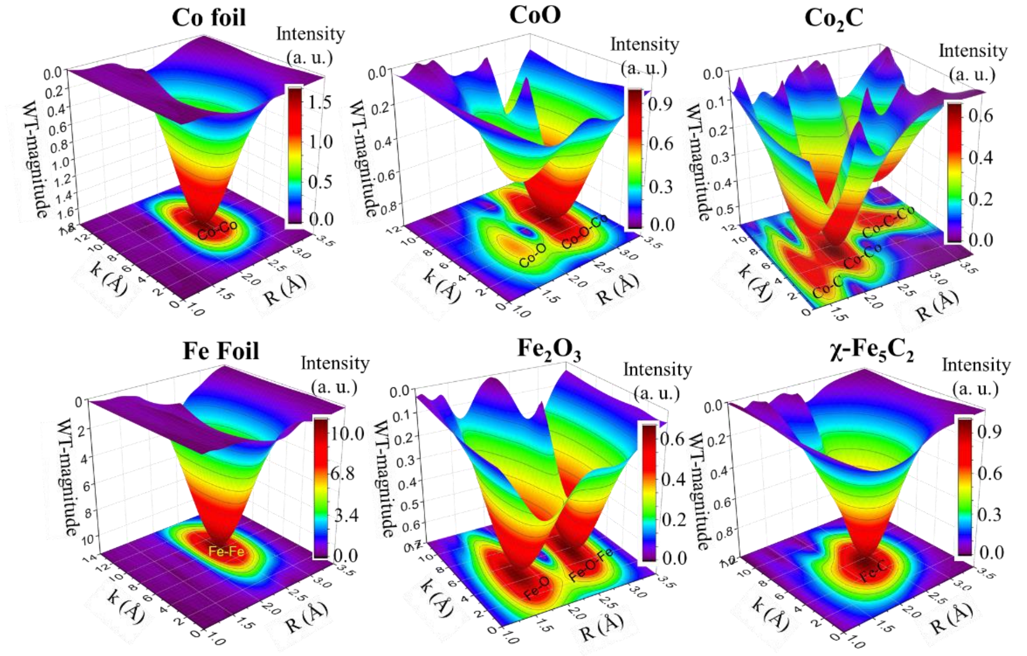

Figure S12 Co K edge WTEXAFS of reference Co foil, CoO, and Co_2C, and Fe K edge WTEXAFS of reference Fe foil, Fe_2O_3, and χ-Fe_5C_2.

Table S2 EXAFS best fitting parameters for Co and Fe K-edge data of the CoFe@FeO_x

Sample	Shell	N	R(Å)	ΔE_0 (eV)	σ^2 (Co-C path)	R-factor（%）
Co	Co-C	2	1.882 ± 0.059	4.49+/- 1.74	3.3+/-1.3	0.0592
	Co-Fe	3	2.368 ± 0.048	4.76+/-1.82	2.8+/-1.1	
Fe	Fe-O	2	1.909 ± 0.059	4.97+/- 1.63	5.5+/-2.3	0.0321
	Fe-C	3	2.283 ± 0.026	3.79+/-2.31	4.9+/-1.8	
	Fe-Co	2	2.589 ± 0.031	2.04+/-1.34	4.8+/-2.5	

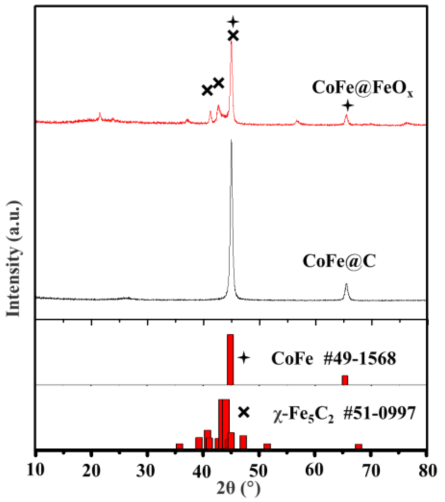

Figure S13 XRD patterns of the CoFe@C and CoFe@FeO_x.

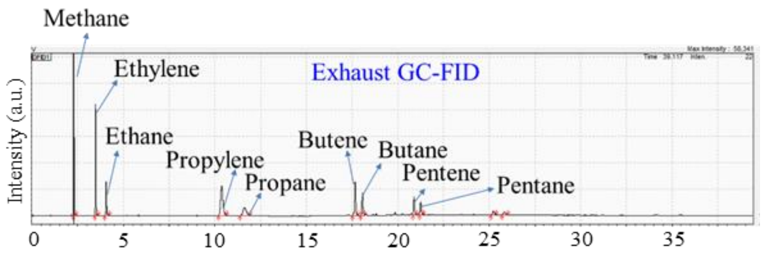

Figure S14 The GC-FID results of the exhaust during carburization.

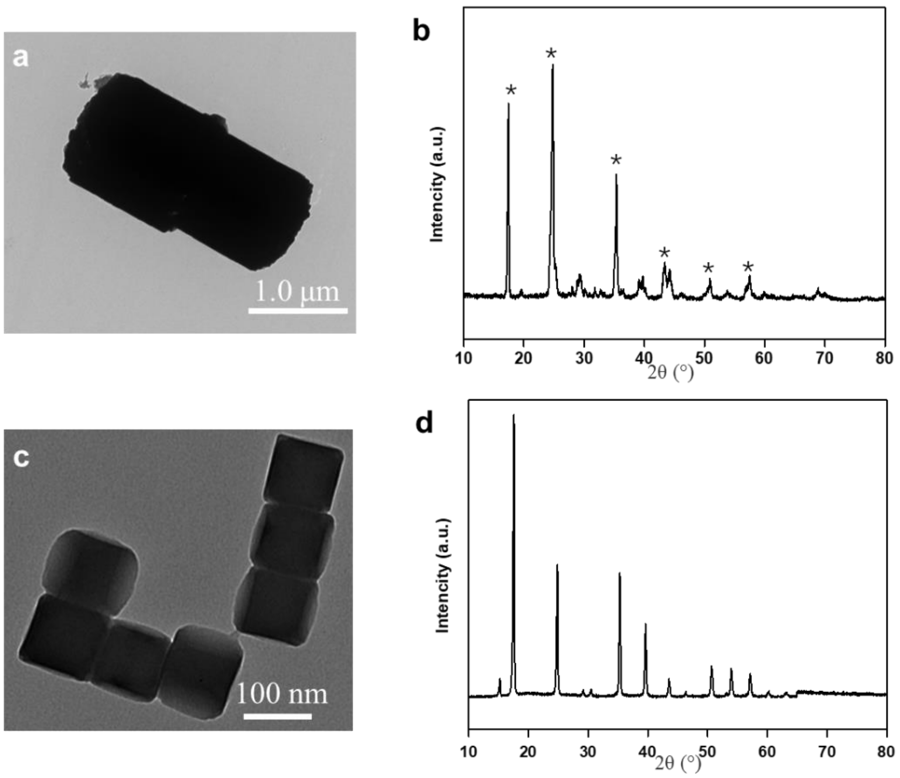

Figure S15 Characterization of Mn-Fe PBA and Ni-Fe PBA. a TEM image and **b** XRD patterns of Mn-Fe PBA. **c** TEM image and **d** XRD patterns of Ni-Fe PBA.

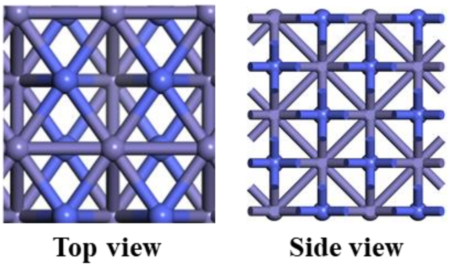

Figure S16 The side and top views of CoFe (110) surface. The blue and purple color balls are Co and Fe atoms, respectively.

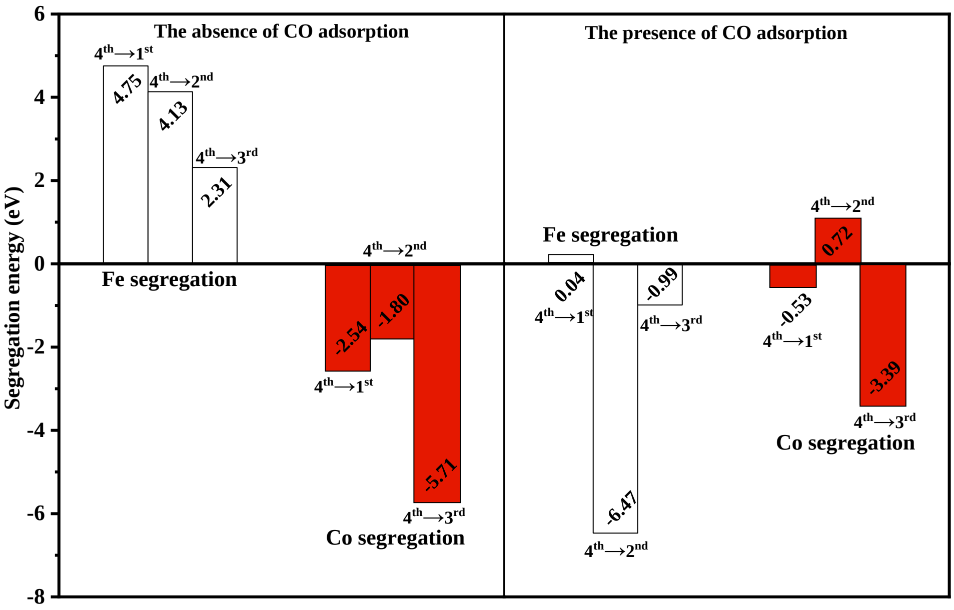

Figure S17 The segregation energy of Fe and Co atoms for the CoFe alloy in the absence and presence of CO adsorption, and the corresponding structures are presented in Figure S16.

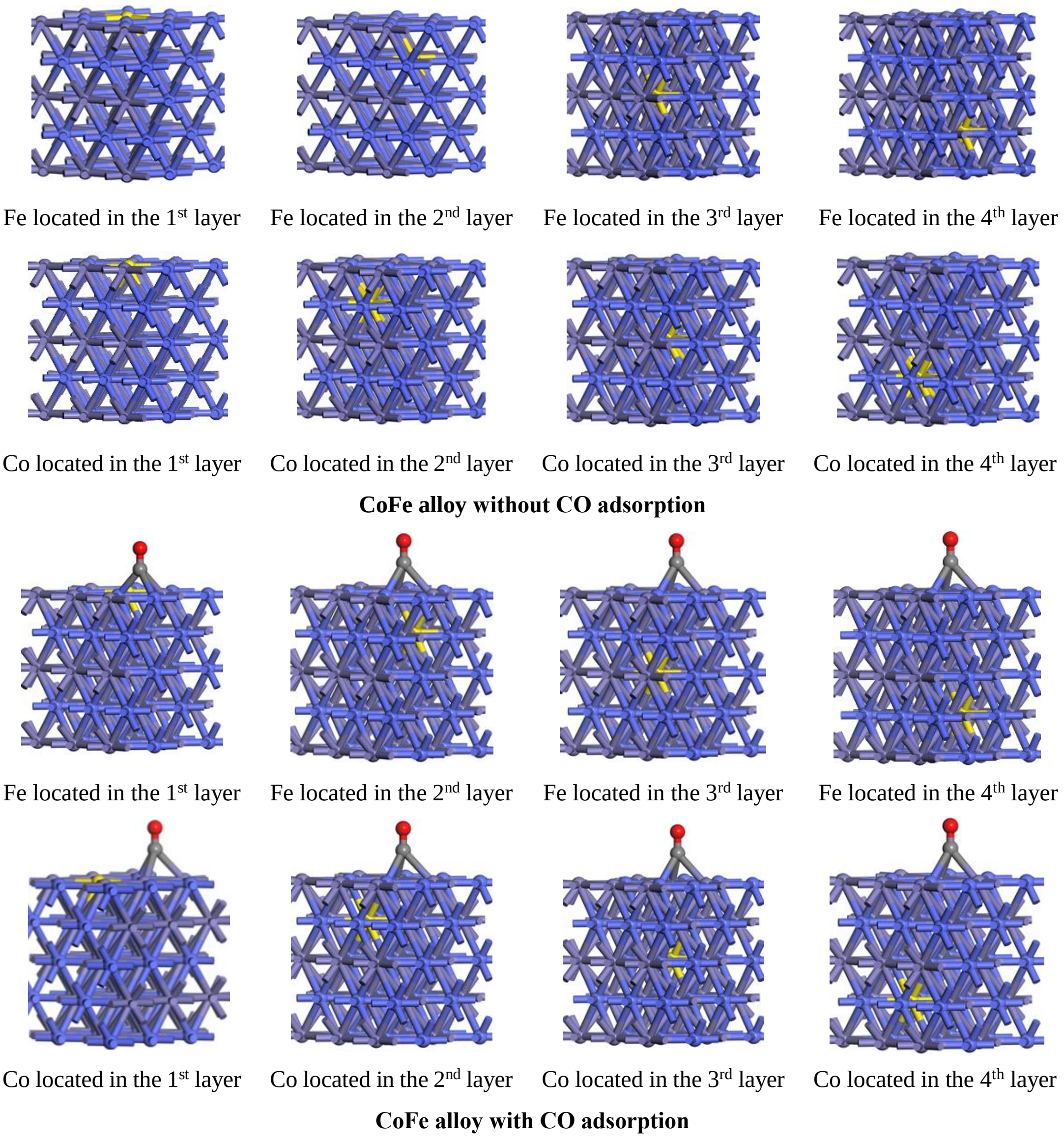

Figure S18 The structures of Fe and Co atoms segregation for the CoFe alloy in the absence and presence of CO adsorption. The blue, purple, grey, and red color balls are Co, Fe, C and O atoms, respectively. The bright yellow represents the Fe or Co atom located in the nth layer.

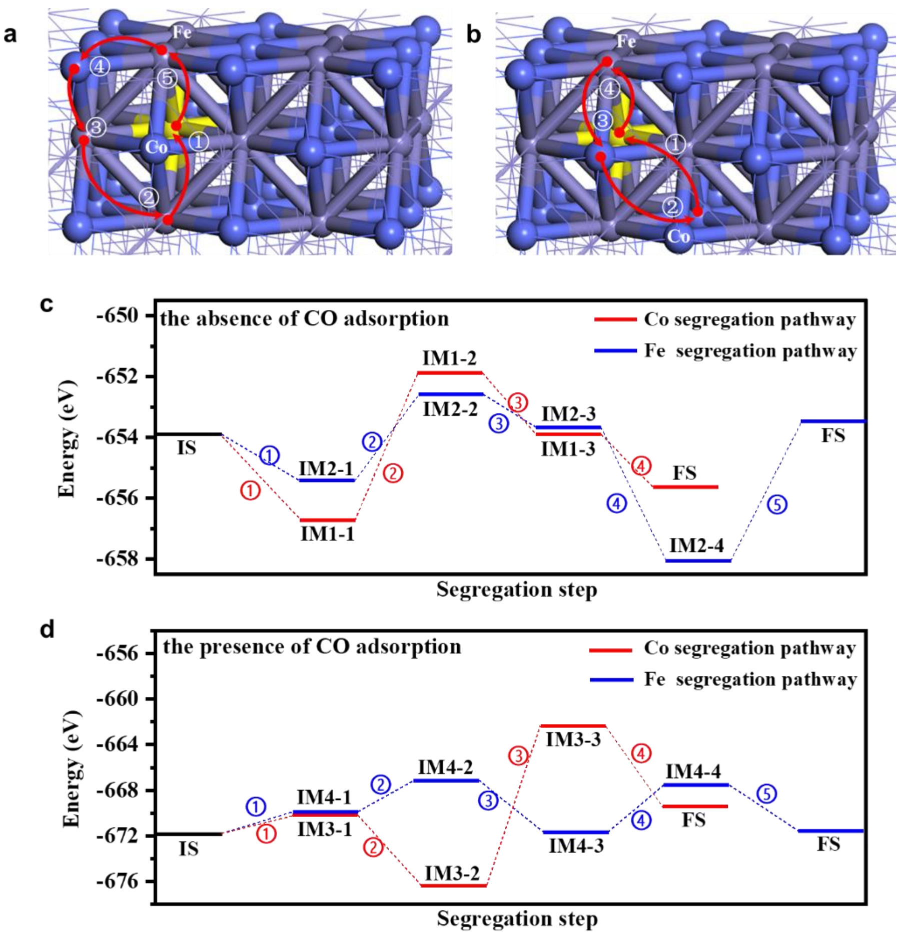

Figure S19 The segregation pathway and reaction energy profile of Fe or Co atom in the CoFe alloy. The segregation pathway of **a** Fe atom and **b** Co atom in the CoFe alloy with the Co vacancy located in the 2nd layer. **c** The reaction energy profile of the Fe or Co atom segregation pathway in the absence of CO adsorption. **d** The reaction energy profile of the Fe or Co atom segregation pathway in the presence of CO adsorption. IS, IM and FS represent the initial, intermediate, and final state, respectively. The blue and purple color balls are Co and Fe atoms, respectively. And the corresponding structures are presented in Figure S18.

In fact, previous studies have confirmed that B2 CoFe alloy prepared by the mechanical alloying showed the presence of vacancy, antisite and point defects.[1-5] *For example, Mizuno et al.*[1] *found that B2 CoFe alloy existed in a wide range of Fe composition from 30 to 75 at% at 773 K, however, aiming at compensating the deviation from the stoichiometric composition, constitutional defects are introduced; the formation energies of the vacancy and antisite defect in the CoFe alloy with different Fe compositions showed that both the vacancy and antisite defect are prone to be formed in the CoFe alloy with 50 at% Fe. Meanwhile, the chemically synthesized B2 CoFe nanoparticle also has defect owing to its synthesis method of one pot polyol process using ethylene glycol as a reducing agent, resulting in the disordered nature.*[2] *Moreover, the vacancies are also experimentally observed in the FeCo alloy, and the self-diffusion of the metals in both the disordered (A2) and ordered (B2) phase CoFe alloy occurs through the vacancies.*[3] *Furthermore, Fu et al.*[4] *theoretically studied the structural stability, point defects and order-disorder transition of B2 CoFe alloy, suggesting that B2 CoFe alloy is marginally stable, weakly ordered with a high density of*

antisite defects. Neumayer et al.[5] *concluded the presence of vacancies in the CoFe alloy based on ab initio statistical mechanics. Above these previously reported studies showed the presence of vacancies in the CoFe alloy, as a result, in our present study, B2 CoFe alloy is employed to explore its Fe segregation, in which B2 CoFe alloy with the vacancy is considered.*

On the other hand, the vacancies in the CoFe alloy cannot be well characterized experimentally in our studies, however, our experiment results showed that Fe atoms would aggregate on the CoFe alloy surface, which means that Fe atoms easily segregates from the bulk to the surface in the CoFe alloy, however, in order to take place the segregation of Fe atoms from the bulk to the surface, only the presence of vacancies in the CoFe alloy could initiate Fe segregation, and realize the segregation of Fe atoms from the bulk to the surface. Moreover, theoretical calculation models of CoFe alloy also further verified that only the presence of vacancies in the CoFe alloy could realize the occurrence of Co/Fe segregation pathway from the bulk to the surface.

Furthermore, the alloy segregation takes place the exchanges between metal ions and surface/subsurface vacancies.[6-8] *For example, Kim et al.*[6] *theoretically found that Au vacancy greatly accelerated the exchanges between Pd and Au in the PdAu alloy, meanwhile, surface Pd segregation induced by CO adsorption on the PdAu alloy surface would become more prominent. DFT studies by An et al.*[7] *investigated Pd surface segregation in the AuPd alloy with the presence of CO, suggesting that Au vacancy is beneficial for promoting the exchanges between Pd and Au, leading to Pd surface segregation. Moreover, Zhang et al.*[8] *found that NiAu core-shell structure exhibited a highly selective CO production in CO_2 hydrogenation due to the formation of a transient reconstructed NiAu alloy surface, in which Ni atoms offer active sites for CO_2 hydrogenation and the surface Au atoms contribute to the selective production of CO; meanwhile, aiming at analyzing the reconstruction of NiAu alloy surface, DFT calculations are adopted to consider the segregation pathway of Ni in the NiAu alloy, then, the model of NiAu alloy is constructed, in which an Au vacancy was set initially at the second atomic layer owing to the easy formation of Au vacancy, and the possible segregation pathway for a third-layer Ni atom near the vacancy was investigated to change the position of Ni through a series of the exchange steps between Au/Ni atom and the vacancy exchange steps.*

Based on above analysis, the presence of vacancies in the CoFe alloy was confirmed, meanwhile, similar to above reported studies by Zhang et al.,[8] *in our present study, a Fe or Co atom vacancy in the CoFe alloy was set initially at the second atomic layer, then, the possible pathway for a third-layer Fe or Co atom near the vacancy was proposed to change the position of Fe or Co atom through a series of the exchange steps between Fe/Co atom and the vacancy, which could realize Fe/Co atom segregation.*

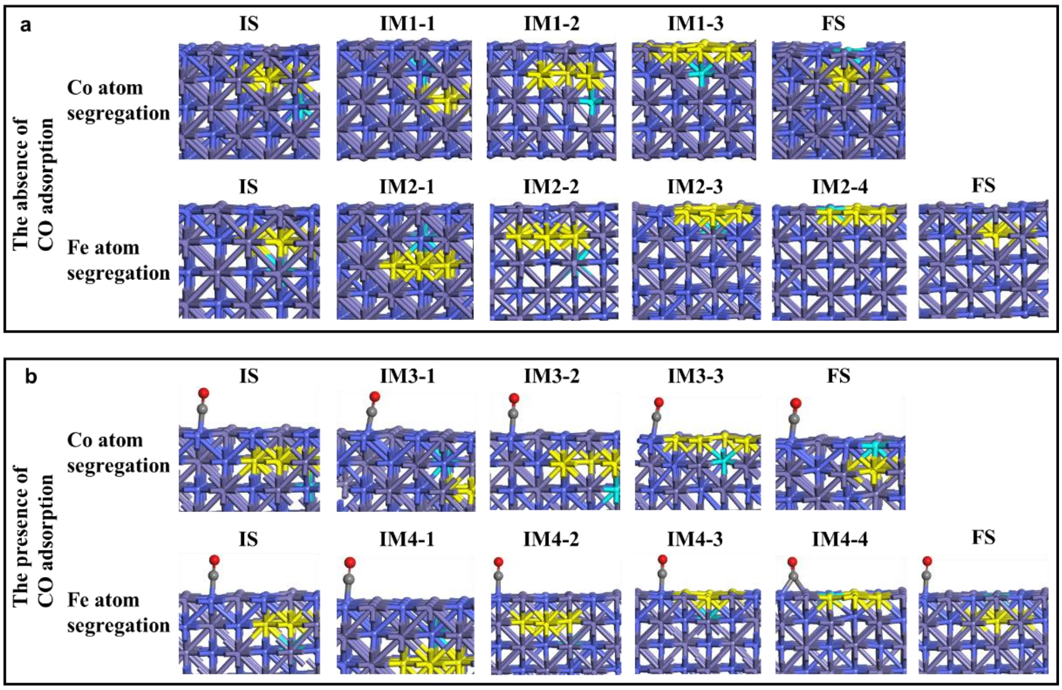

Figure S20 The Fe and Co atoms segregation pathway in the CoFe alloy. The structures of Fe and Co atoms segregation pathway in the **a** absence or **b** presence of CO adsorption in the CoFe alloy with Co vacancy located in the 2nd layer. IS, IM and FS represent the initial, intermediate, and final state, respectively. The blue, purple, grey, and red color balls are Co, Fe, C and O atoms, respectively. The bright yellow represents the vacancy located in the nth layer. The bright blue represents the M (M=Fe or Co atom) located in the nth layer.

For Fe atoms segregation in the CoFe alloy, as shown in Figures S19 and S20, in the absence of CO adsorption, the E_{seg-1}, E_{seg-2} and E_{seg-3} of Fe atom for CoFe alloy are 4.75, 4.13 and 2.31 eV, respectively, suggesting that the ability of Fe atom segregation from the bulk to the surface is weaker. Meanwhile, the E_{seg-1}, E_{seg-2} and E_{seg-3} of Co atom for CoFe alloy are -2.54, -1.80 and -5.71 eV, respectively, which means that the Co atom segregation from the bulk to the surface for CoFe alloy is much easier compared to the Fe atom segregation in the absence of CO adsorption. In the presence of CO adsorption, CO adsorption alters the $E_{seg-n(n=1-3)}$ of Fe and Co atoms for CoFe alloy. The E_{seg-1}, E_{seg-2} and E_{seg-3} of Fe atom are 0.04, -6.47 and -0.99 eV, respectively, indicating that the Fe atom segregation from the bulk to the surface easily occurs. However, for Co atom segregation from the bulk to the surface, the E_{seg-1}, E_{seg-2} and E_{seg-3} of Co atom are -0.53, 0.72 and -3.39 eV, respectively. Thus, the segregation of Fe atom is much easier than the segregation of Co atom in the presence of CO adsorption. Above results show that compared to the segregation of Fe or Co atom in the CoFe alloy in the absence of CO adsorption, CO adsorption promotes the segregation of Fe atom; while it suppresses the segregation of Co atom. As a result, under CO atmosphere, the presence of CO adsorption promotes the Fe atom segregation in the CoFe alloy instead of the Co atom segregation, and the CoFe alloy presents the enriched surface Fe atoms, which are preferentially carburized to form a Fe_xC shell on the CoFe alloy.

Aiming at further analyzing the preference of Fe or Co atom segregation in the CoFe alloy, the reaction energy of Fe or Co atom segregation pathway is calculated. As mentioned above, in general, the alloy segregation takes place with the exchanges between the metal ions and

surface/subsurface vacancies[6-8]*. Our results show that the formation energies of Fe or Co vacancy initially set in the 2nd layer with the absence of CO adsorption are 5.5 or 2.0 eV, namely, the formation of Co vacancy in the 2nd layer for CoFe alloy more easily occurs compared to that of Fe vacancy. Further, compared to the CoFe alloy surface in the absence of CO adsorption, CO adsorption promotes the formation of Fe or Co vacancy in the 2nd layer for CoFe alloy, however, the formation energy of Co vacancy is lower than that of Fe vacancy (-16.0 vs. -14.3 eV), namely, the formation of Co vacancy is much easier than that of Fe vacancy. Above results show that the formation of Co vacancy for the CoFe alloy is easier than that of Fe vacancy irrespective of CO adsorption, thus, only the Co vacancy in the 2nd layer for CoFe alloy is considered in our study.*

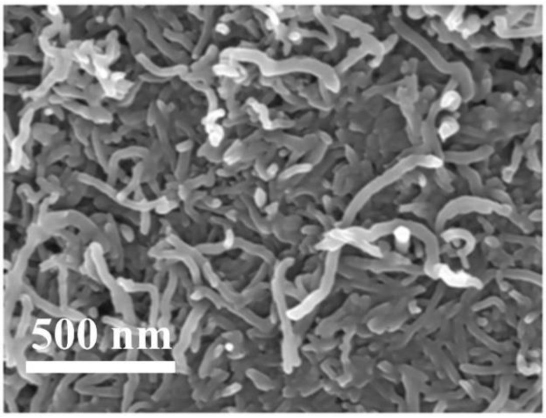

Figure S21 The commercial multiwalled CNTs and S powder composite cathode (S content: 70 wt%).

Commercial multiwalled CNTs and S powder composite is employed as cathode, in which active S accounts for 70 wt% in the composite cathode as shown in Figure S21.

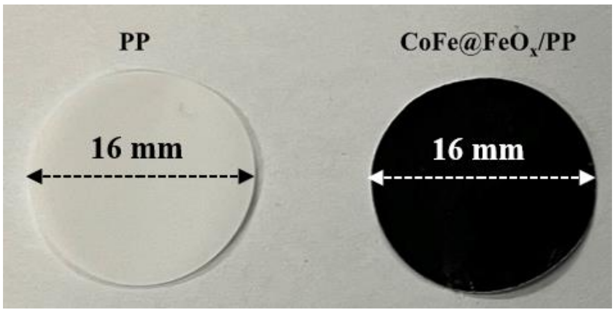

Figure S22 The optical photographs of the commercial PP separator and CoFe@FeO_x/PP separator.

The CoFe@FeO_x/PP obtained via the tape casting exhibits smooth surface according to the optical photographs in Figure S22, which is similar to that of commercial PP separator.

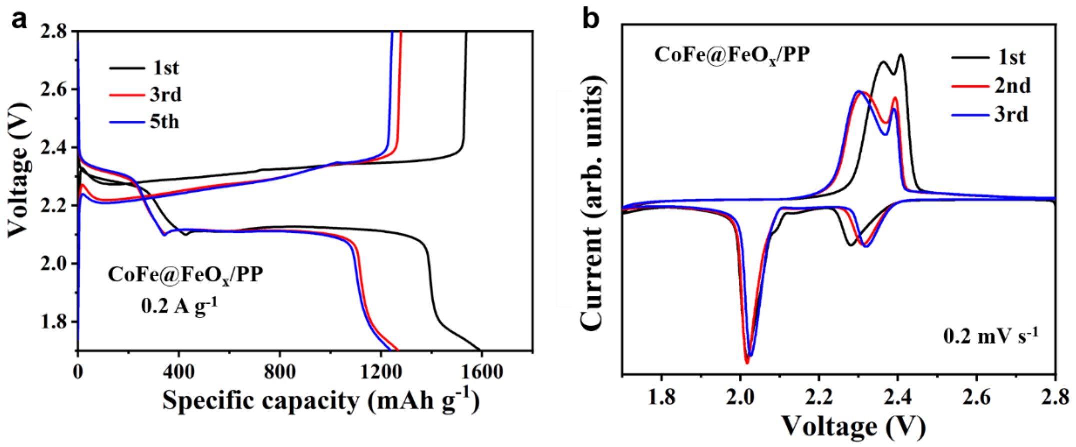

Figure S23 Electrochemical tests of Li-S batteries with CoFe@FeO$_x$/PP with 70 wt% of sulfur loading in the cathode. **a** Typical charge/discharge curves at 0.2 A g^{-1}. **b** Typical cyclic voltammogram (CV) curves at 0.2 mV s^{-1}.

The charge/discharge curves of Li-S battery are shown in Figure S23a. The battery with CoFe@FeO$_x$/PP can deliver a high initial capacity of 1537 mAh g^{-1} (around 92% of theoretical value) at 0.2A g^{-1}. According to CV curves at 0.2 mV/s (Figure S23b), two typical cathodic peaks can be assigned to the sequential reduction of S_8 to soluble LiPSs and further convert to solid Li_2S_2/Li_2S. The charge plateau at 2.23-2.42 V is related to oxidation reaction of Li_2S to LiPSs and S_8.

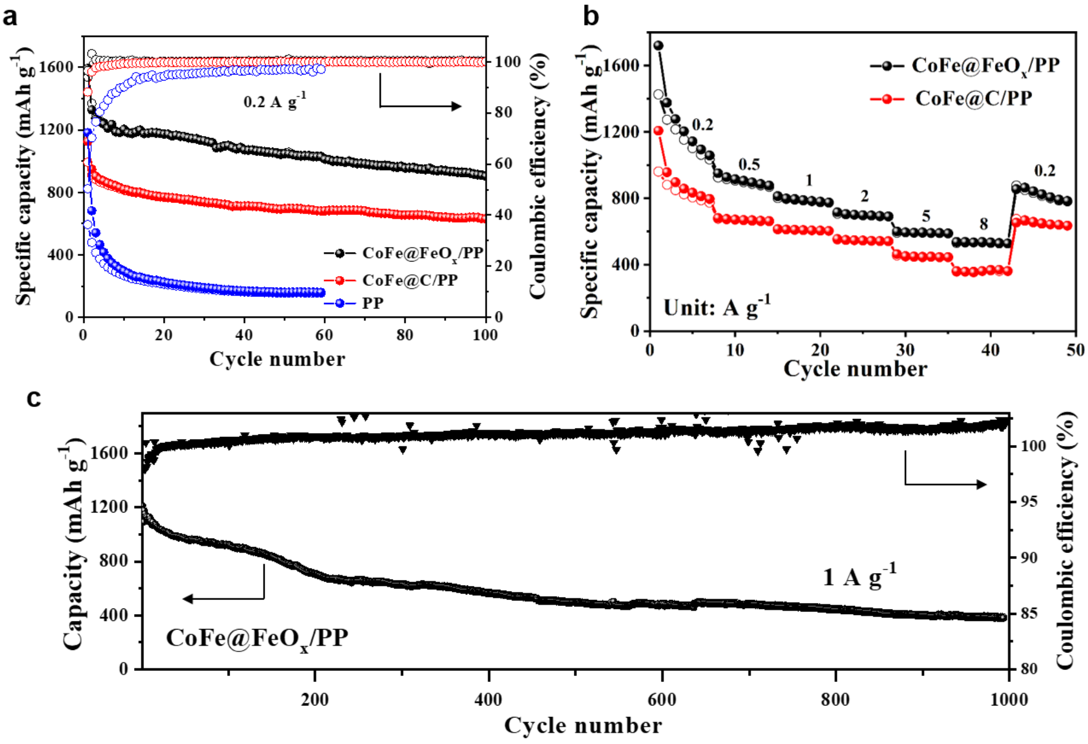

Figure S24 Electrochemical performance with different separators. **a** Cycling performance comparison of Li-S batteries with different separators with 70 wt% of sulfur loading in the cathode at 0.2 A g^{-1}. **b** Rate capability comparison of CoFe@FeO$_x$/PP and CoFe@C/PP based Li-S batteries with 70 wt% of sulfur loading in the cathode at various current densities. **c** Long-term cycling life of CoFe@FeO$_x$/PP based Li-S batteries with 70 wt% of sulfur loading in the cathode at 1 A g^{-1}. The

batteries in Figure **S24** a-c are composed of Na metal anode and CNTs and S composite cathode with 70 wt% of sulfur loading.

Detailed performance comparisons of Li-S batteries with traditional PP separator, CoFe@C/PP and CoFe@FeO$_x$/PP are exhibited in Figure S24a. The Li-S battery with CoFe@FeO$_x$/PP can maintain a high reversible capacity of 913 mAh g^{-1} after 100 cycles. As a contrast, a low initial capacity of 595 mAh g^{-1} is obtained with the traditional PP separator, demonstrating the CoFe@FeO$_x$ can effectively anchor LIPSs and facilitate the conversion of sulfur species. And the Li-S battery with the CoFe@C/PP exhibits an initial reversible capacity of 993 mAh g^{-1} at 0.2 A g^{-1} and maintain a reversible capacity of 631 mAh g^{-1} after 100 cycles, which is poorer than that of the CoFe@FeO$_x$ based battery. The rate performance comparison of batteries with the CoFe@FeO$_x$/PP and CoFe@C/PP are showed in Figure S24b. The CoFe@FeO$_x$ based battery can exhibit high specific capacities of 1376, 927, 799, 706, 594 and 536 mAh g^{-1} at 0.2, 0.5, 1, 2, 5 and 8 A g^{-1}, respectively. However, the battery with CoFe@C/PP could only deliver specific capacities of 957, 675, 612, 549, 451 and 358 mAh g^{-1} at 0.2, 0.5, 1, 2, 5 and 8 A g^{-1}, respectively. The long-term cycling performance with the CoFe@FeO$_x$/PP is evaluated at 1 A g^{-1} as shown in Figure S24c, which could deliver a long lifespan around 1000 cycles. The superior electrochemical performance suggests that the CoFe@FeO$_x$ with abundant polar active sites in the FeO$_x$ shell and highly conductive CoFe alloy core can significantly suppress the shuttling of polysulfides and promote the conversion process of LIPSs.

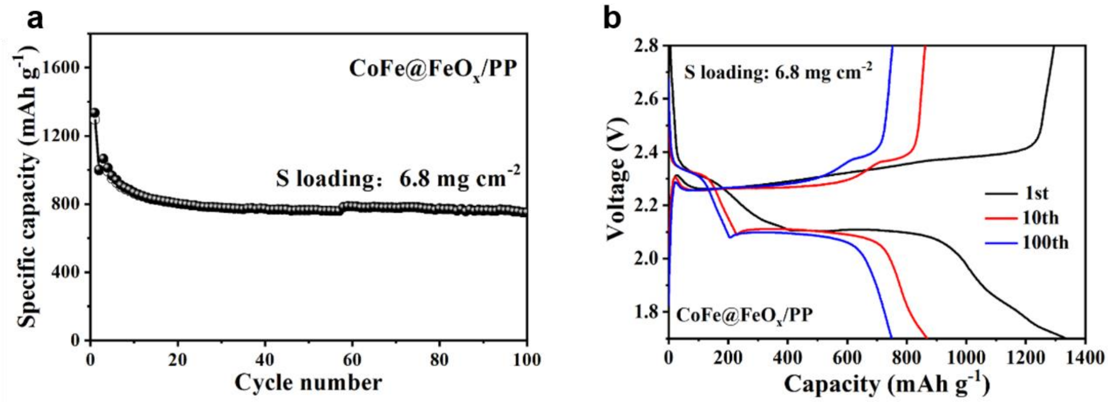

Figure S25 Electrochemical performance of CoFe@FeO$_x$/PP based battery with high sulfur loading of 6.8 mg cm^{-2} at 0.1 A g^{-1}. a Cycling performance. **b** Corresponding charge/discharge curves This battery is composed of Na metal anode and CNTs and S composite cathode with 70 wt% of sulfur loading.

The high areal S loading electrode is fabricated to further check practical application of Li-S batteries with the CoFe@FeO$_x$/PP (Figure S25a and S25b). Even with high sulfur loading mass of 6.8 mg cm^{-2}, the CoFe@FeO$_x$ based battery can achieve a high initial capacity of 1294 mAh g^{-1} and maintain a superior reversible capacity of 752 mAh g^{-1} after 100 cycles at 0.1A g^{-1}, indicating the desirable application prospect of CoFe@FeO$_x$ for boosting high energy density practical Li-S batteries.

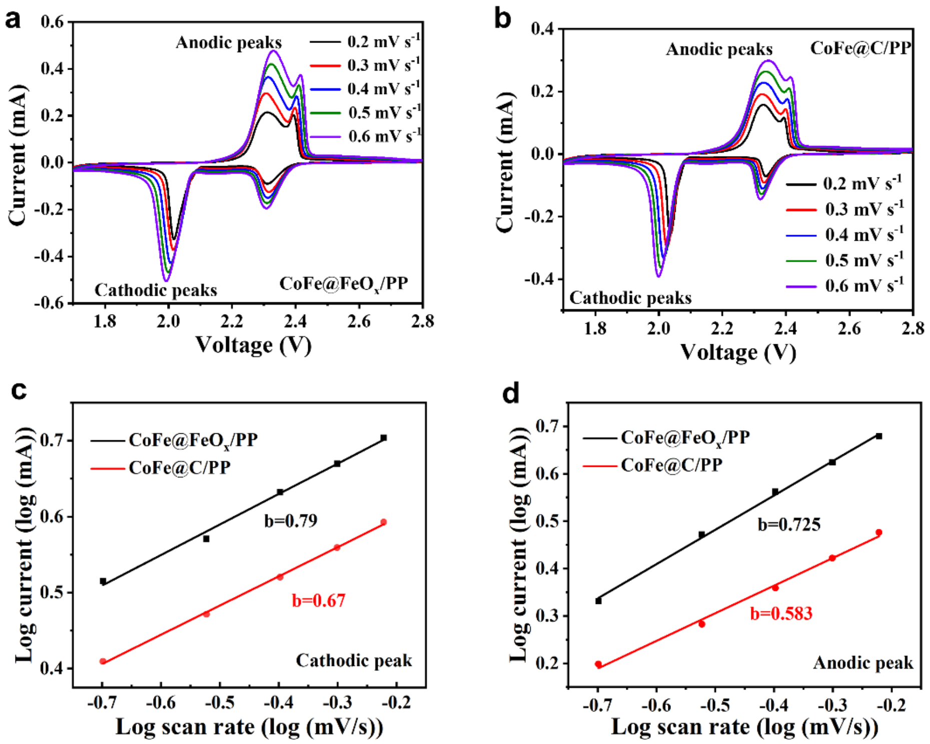

Figure S26 CV curves and corresponding linear fitting plots. CV curves of **a** CoFe@FeO$_x$/PP and **b** CoFe@C/PP based Li-S batteries with 70 wt% of sulfur loading in the cathodes at various scan rates. The linear fitting plots of **c** CoFe@FeO$_x$/PP and **d** CoFe@C/PP based batteries.

As shown in Figure S26a-d, the reaction dynamics comparisons between the CoFe@FeO$_x$ and CoFe@C based batteries are analyzed via the power-law equation (log(i) = log(a) + blog(v), i and v are the peak current and scanning rate and a and b are the adjustable parameters).[23] *Both the cathodic peak and anodic peak with the CoFe@FeO$_x$ based battery show higher b values than those of the CoFe@C based battery, demonstrating much faster reaction kinetics with the CoFe@FeO$_x$/PP.*

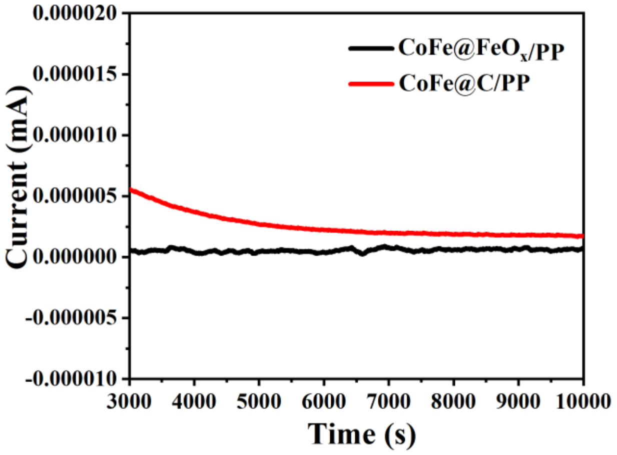

Figure S27 The shuttle current measurement for Li-S batteries with CoFe@FeO$_x$/PP and CoFe@C/PP.

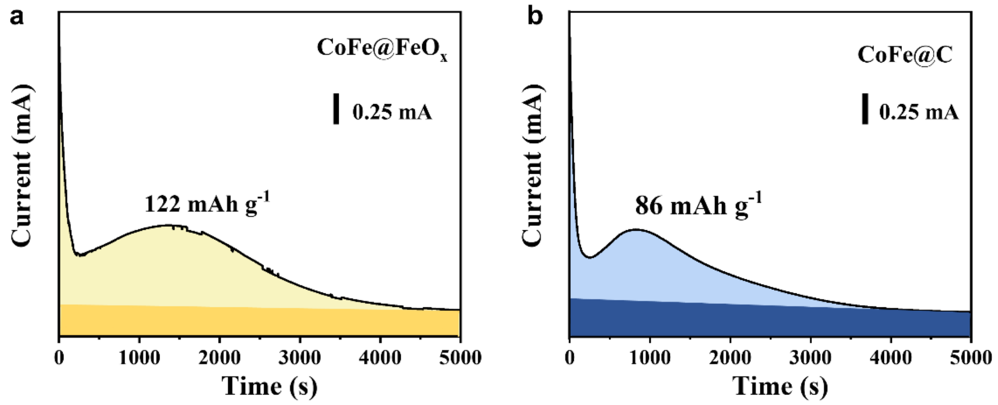

Figure S28 The Li_2S precipitate experiments for Li-S batteries. a CoFe@FeO_x. b CoFe@C.

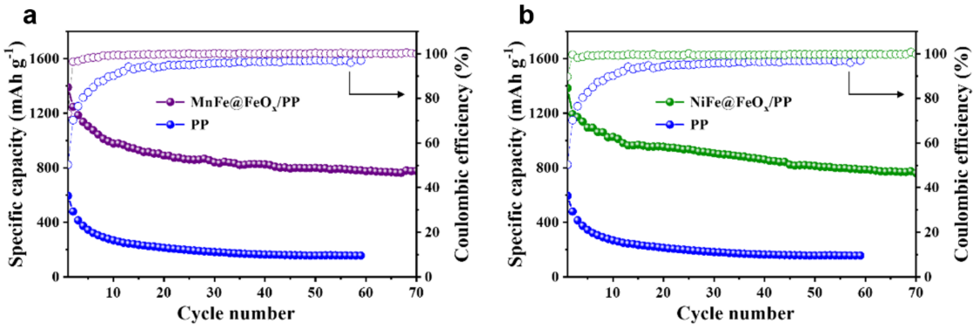

Figure S29 Cycling performance with different separators. Cycling performance of **a** MnFe@FeO_x/PP and **b** NiFe@FeO_x/PP based Li-S batteries at 0.2 A g^{-1}. The batteries in **Figure S29** are composed of Na metal anode and CNTs and S composite cathode with 70 wt% of sulfur loading.

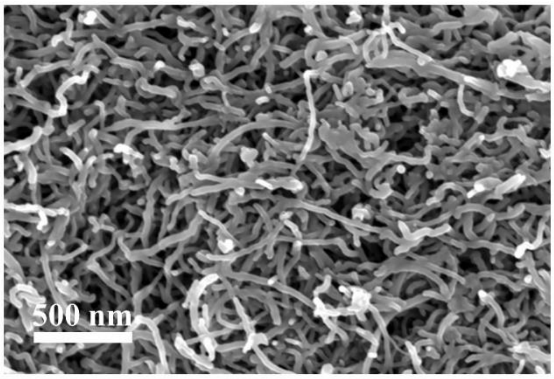

Figure S30 The commercial multiwalled CNTs and S powder composite cathode (S loading: 50 wt%).

In view of more slower reaction kinetics derived from larger Na ion radius and more severe volume expansion issues of Na-S batteries than those of Li-S batteries, the CNTs and S composite cathode with S content of 50 wt% is attempted to assemble Na-S batteries. The CNTs and S composite cathode with good dispersion is shown in Figure S30.

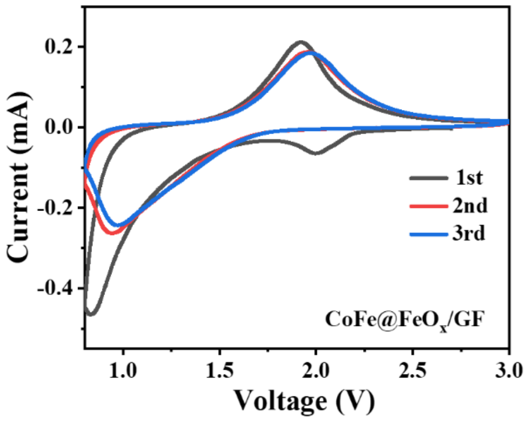

Figure S31 The typical CV curves of Na-S battery with CoFe@FeO_x/GF with 50 wt% of sulfur loading at 0.2 mV s^{-1}.

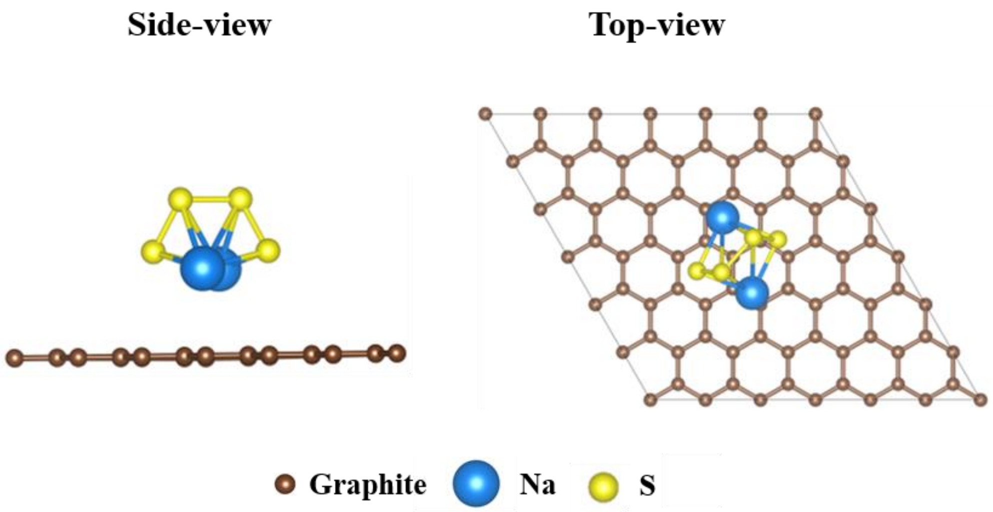

Figure S32 Optimized adsorption configuration of Na_2S_4 on the surface of graphite.

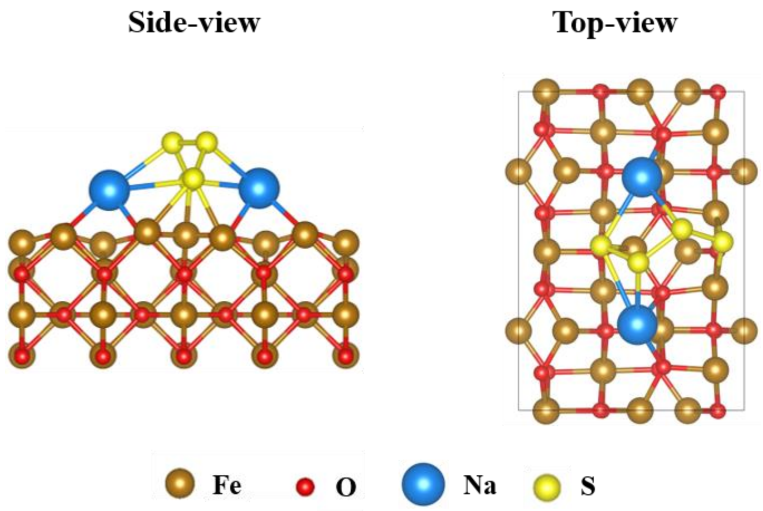

Figure S33 Optimized adsorption configuration of Na_2S_4 on the surface of Fe_3O_4.

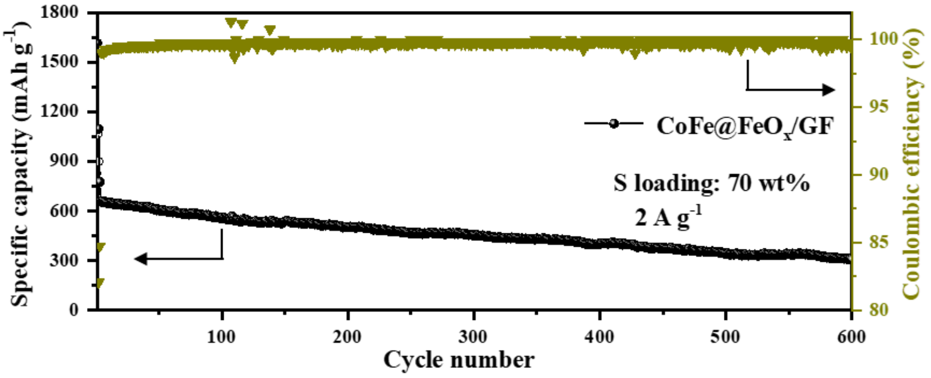

Figure S34 Cycling performance of CoFe@FeO$_x$/GF based Na-S batteries at 2 A g^{-1}. This battery is composed of Na metal anode and CNTs and S composite cathode with 70 wt% of sulfur loading.

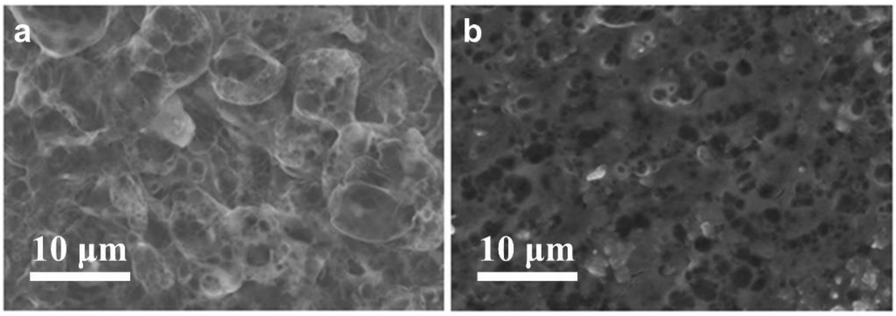

Figure S35 Morphology analysis. The morphologies of Na anode for Na-S batteries with 50 wt% of sulfur loading with **a** commercial GF separator and **b** CoFe@FeO$_x$/GF separator after three cycles at 0.2 A g^{-1}.

The morphologies of Na anode with commercial GF separator and CoFe@FeO$_x$/GF after three cycles at 0.2 A g^{-1} are displayed in Figure S35a and S35b. Na anode exhibits rough and corroded surface in Na-S battery with the commercial GF separator. When used the CoFe@FeO$_x$/GF separator, the Na anode presents relatively smooth surface, further demonstrating the CoFe@FeO$_x$/GF separator could inhibit the shuttle effect and protect the Na anode.

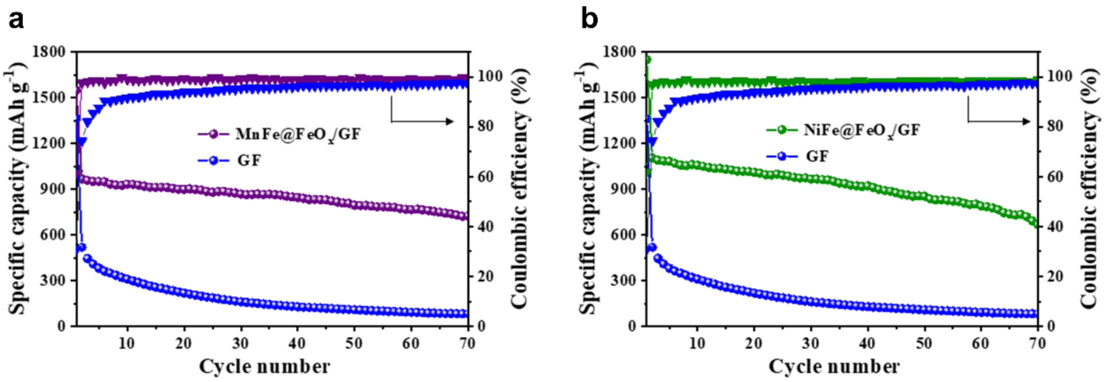

Figure S36 Cycling performance with different separators. Cycling performance of **a** MnFe@FeO$_x$/GF and **b** NiFe@FeO$_x$/GF based Na-S batteries at 0.2 A g^{-1}. The batteries in **Figure**

S36 are composed of Na metal anode and CNTs and S composite cathode with 50 wt% of sulfur loading.

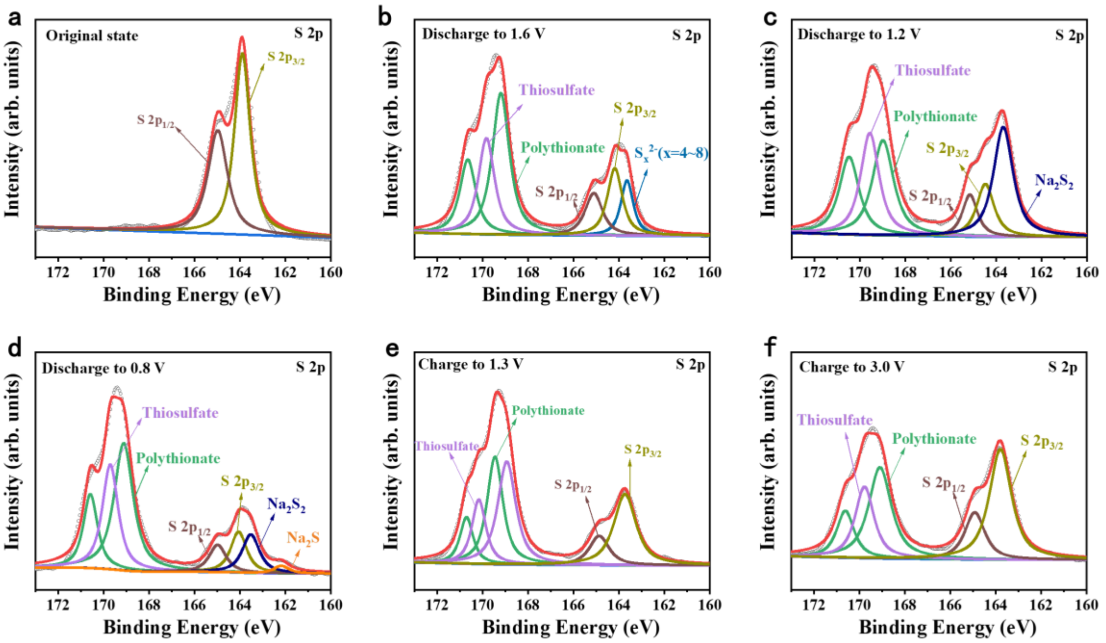

Figure S37 ***Ex-situ*** **XPS spectra of the CoFe@FeO$_x$/GF based Na-S battery with 50 wt% of sulfur loading in the cathode measured at different discharge and charge states.** **a** Original state, **b** Discharge to 1.6 V, **c** Discharge to 1.2 V, **d** Discharge to 0.8 V, **e** Charge to 1.3 V and **f** Charge to 3.0 V.

References

1. Mizuno, M. et al. First Principles Calculation of Defect and Magnetic Structures in FeCo. *Mater. Trans.* **47**, 2646−2650 (2006).
2. Rajesh, P. et al. Positron annihilation studies on chemically synthesized FeCo alloy. *Sci. Rep. UK* **8**, 9764 (2018).
3. Seeger, A. Ordering processes and atomic defects in FeCo. *Int. J. Mater. Res.* **97**, 861−871 (2022).
4. Fu, C. et al. First-principles study of the structural, defect, and mechanical properties of B2 FeCo alloys. *Phys. Rev. B* **74**, 174108 (2006).
5. Neumayer, M. et al. Atomic defects in FeCo: Stabilization of the B2 structure by magnetism. *Phys. Rev. B* **64**, 132102 (2001).
6. Kim, H. et al. CO Adsorption-driven surface segregation of Pd on Au/Pd bimetallic surfaces: Role of defects and effect on CO oxidation. *ACS Catal.* **3**, 2541−2546 (2013).
7. An, H. et al. Understanding the atomic-level process of CO-adsorption-driven surface segregation of Pd in $(AuPd)_{147}$ bimetallic nanoparticles. *Nanoscale* **9**, 12077−12086 (2017).
8. Zhang, X. et al. Reversible loss of core–shell structure for Ni–Au bimetallic nanoparticles during CO_2 hydrogenation. *Nat. Catal.* **3**, 411−417 (2020).
